# Reduction of Hexavalent Chromium and Detection of Chromate Reductase (ChrR) in *Stenotrophomonas maltophilia*

**DOI:** 10.3390/molecules23020406

**Published:** 2018-02-13

**Authors:** Rosa Baldiris, Natali Acosta-Tapia, Alfredo Montes, Jennifer Hernández, Ricardo Vivas-Reyes

**Affiliations:** 1 Grupo de Microbiología Clínica y Ambiental, Facultad de Ciencias Exactas y Naturales, Programa de Biología, Universidad de Cartagena, Campus San Pablo, Cartagena 130015, Colombia; nacostat2@unicartagena.edu.co (N.A.-T.); alfredolaroa@gmail.com (A.M.); 2 Grupo de Investigación CIPTEC, Facultad de Ingeniería, Programa de Ingeniería de Procesos, Fundación Universitaria Tecnológico Comfenalco, Cartagena 130015, Colombia; rvivasr@unicartagena.edu.co; 3 Grupo de Química Cuántica y Teórica, Facultad de Ciencias Exactas y Naturales, Universidad de TCartagena, Campus, San Pablo, Cartagena 130015, Colombia; jheniferhernandez29@gmail.com

**Keywords:** *Stenotrophomonas maltophilia*, chromate reductase, *ChrR*, Cr(VI), heavy metal

## Abstract

An Gram negative strain of *S. maltophilia*, indigenous to environments contaminated by Cr(VI) and identified by biochemical methods and 16S rRNA gene analysis, reduced chromate by 100%, 98–99% and 92% at concentrations in the 10–70, 80–300, and 500 mg/L range, respectively at pH 7 and temperature 37 °C. Increasing concentrations of Cr(VI) in the medium lowered the growth rate but could not be directly correlated with the amount of Cr(VI) reduced. The strain also exhibited multiple resistance to antibiotics and tolerance and resistance to various heavy metals (Ni, Zn and Cu), with the exception of Hg. Hexavalent chromium reduction was mainly associated with the soluble fraction of the cell evaluated with crude cell-free extracts. A protein of molecular weight around 25 kDa was detected on SDS-PAGE gel depending on the concentration of hexavalent chromium in the medium (0, 100 and 500 mg/L). In silico analysis in this contribution, revealed the presence of the chromate reductase gene *ChrR* in *S. maltophilia*, evidenced through a fragment of around 468 bp obtained experimentally. High Cr(VI) concentration resistance and high Cr(VI) reducing ability of the strain make it a suitable candidate for bioremediation.

## 1. Introduction

Cr is a heavy metal belonging to the transition group (VI-B) of the modern periodic table. Though, it exists in several oxidation states ranging from −2 to +6 due to its electronic structure, Cr(III) and Cr(VI) are the most common chromium compounds, both having different physicochemical and biochemical properties [1,2].

Cr(III) is 10–100 times less bioavailable, relatively stable and concurrently less toxic than Cr(VI) [3]. Cr(VI) is considered the most toxic form of Cr due to its strong oxidation potential, higher solubility in water, rapid permeability through biological membranes and it has recently been classified as one of the 17 most toxic chemicals by the Agency for Toxic Substances and Diseases Registry (ATSDR) and listed as grade ‘A’ human carcinogen by the United States Environmental Protection Agency (U.S. EPA) [4,5]. Cr(VI) is a well-established occupational carcinogen associated with lung cancer and nasal and sinus cancer. NIOSH considers all Cr(VI) compounds to be occupational carcinogens.

In the carcinogenic behavior of chromium, chromate (CrO_4_^2−^) (a strong oxidizing agent) is reduced intracellularly to Cr(V) which forms reactive species and free radicals that could damage DNA and other biomolecules producing a wide spectrum of genomic alterations such as DNA strand breaks, alkali-labile sites, DNA-protein, and DNA-DNA crosslinks, and Cr(III)-DNA adducts associated with the mutagenic effects in biological systems [6,7,8].

Chromate compounds have a lot of uses, for instance, chromate (CrO_4_^2−^), and dichromate, (Cr_2_O_7_^2−^), are use as oxidizing agents. They are used in laboratories to clean organic material from the surfaces of glassware. This kind of solution should be handled with extreme care because Cr(VI) compounds are a powerful corrosive and carcinogen agents. NIOSH recommends that airborne exposure to all Cr(VI) compounds be limited to a concentration of 0.2 μg Cr(VI)/m^3^ for an 8-h [9]. However, the concentrations during the washing of the material could be higher. This is due to the presence of metal–carbon bonds, several transformations can occur under physiological conditions. Some of this organo-chromium complexes are volatile, which facilitates their distribution in the environment [10].

Hexavalent chromium is used in a variety of industrial applications such as leather tanning, metallurgy, electroplating, petroleum refining, textile dyeing, paints, pigment production, steel making and thermonuclear weapons manufacturing [11,12,13]. Similar to some organic pollutants, chromium tend not to be degraded and accumulated into the environment, chromium compounds may enter to food chain and could cause different effects [14].

Moreover, is a well-established that Cr(VI) released into the environmental and exposure causes serious health problems such as accumulating in the placenta, impairing fetal development, skin allergies, vomiting, diarrhea, brain damage and premature death in mammals [15]. Other reports indicate effects on plants such as metabolic alterations, poor seed germination, stunted root growth, photosynthetic impairment and death. This delicate situation has generated an alert around the world, evidencing the need for efficient treatments for bioremediation of environments contaminated with this metal and for the correct treatment of toxic industrial wastes. Hence the importance of reducing the chromium oxidation state.

Several reports have shown that biological reduction of Cr(VI) by microorganisms and their enzymes is considered one of the most practical and useful methods for reducing Cr(VI) to relatively less toxic Cr(III) [8], being an economical and environmentally friendly [16] as compared to anion exchange resins, electrolysis, among other treatments. Although they can efficiently reduce these compounds they are chemically synthesized, costly and also are not safe [17,18].

There is a lot of evidence that shows that the reduction of Cr(VI) by means of a wide variety of bacteria such as *Bacillus* sp. [19] *Exiguobacterium* sp. [20], *Staphylococcus aureus and Pediococcus pentosaceus* [4], *Stenotrophomonas maltophilia*, *Pantoea* sp., and *Aeromonas* sp. [21] under aerobic and anaerobic conditions or sometimes both. This bacterial capability has been acquired in response to environmental conditions and these chromium-resistant bacteria can perform bioreductions for longer periods [22]. In metal-contaminated environments, they undergo quick mutation to develop Cr(VI) resistance that leads to reduce Cr(VI) uptake through the sulfate transport channel present in the bacterial membranes, due to the structural similarity between these molecules [23], while susceptible organisms can become insensitive by mutation or by incorporation of the genetic information which encodes for the resistance [24].

In chromium-resistant bacteria, different chromate reductases such as ChrR, YieF, NemA and LpDH, catalyze the reduction of Cr(VI) to Cr(III) mediating the transfer of electrons from electron donors like NAD(P)H to Cr(VI) and simultaneous generation of reactive oxygen species (ROS) by two reaction mechanisms, namely, Class I (“tight”) and Class II (“semi-tight”). These enzymes are located either in the soluble fractions (cytoplasm) or bound to the membrane of the bacterial cell. Soluble reductases are suitable for the development of biocatalysts for bioremediation since those are more amenable to protein engineering to suit the environmental conditions of contaminated sites when compared to the membrane-bound chromate reductases [8].

It is well known that tannery industries are among the major sources of chromium contamination into the environment. The Cr concentration in tannery wastewater ranges between 2000 and 5000 mg/L, which is much higher than the permissible limit of 2 mg/L for wastewater discharge [25]. In Colombia, a series of studies have been performed to assess the degree of contamination caused by chromium. These researches, for example in the Bogota River, show that there concentrations above 37.3 mg/kg are present, associated mainly with the leather industry. Currently, a maximum value is accepted of 10 μg/L for the general population and 20 μg/L for the occupationally exposed population. These values are alarming due to their high concentration. In Cartagena, for instance there is a series of home industries that work with chrome in a very rudimentary way, this type of industry works under no control by any environmental authority, resulting in the dumping of these water bodies directly into the sea without any control.

All these issues prompted us to wonder whether these questions could be answered or, at least, clarified by identification de *S.* *maltophilia* and a reduction mediation mechanism for chromate reductases (ChrR), and finding a cellular route that bacteria use to have this advantage over other microorganisms. 

The purpose of this paper was thus to isolate, characterize and screen Cr(VI)-resistant bacteria from residual effluents of auto part chroming processes, evaluate their ability to reduce/detoxify hexavalent chromium and investigate the mechanisms associated with Cr(VI) reduction mediation for chromate reductases (ChrR) by *S.* *maltophilia*.

## 2. Results and Discussion

The bacterial isolate could grow at a pH range of 3.0–12.0, the optimum pH being 7, and temperature range of 28–45 °C, the optimum temperature for growth being 37 °C under 200 rpm in a water bath. A total of 13 morphologically different bacterial isolates were able to grow on plates amended with 100, 300 and 500 mg/L of K_2_Cr_2_O_7_. These isolates were further inoculated in LB broth supplemented with different concentrations of Cr(VI) to determine their Cr(VI) tolerance between 10 and 2000 mg/L.

**Figure 1:**
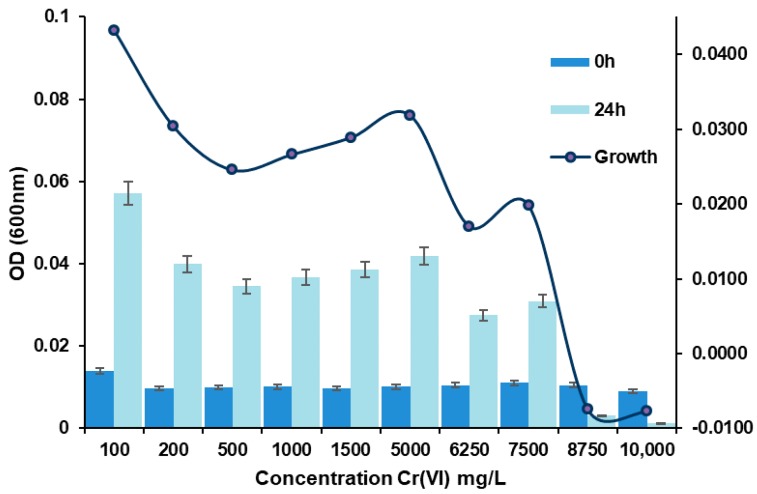
Growth of strain NA2. The cells were cultured on Luria–Bertani broth supplemented with 100, 200, 500, 1000, 1500, 5000, 6250, 7500, 8750 and 10,000 mg/L Cr(VI), respectively. The optical density was measured after incubation for 24 h at 37 °C.

Isolate NA2 showed highest level of tolerance, growing well in presence of 1950 mg/L Cr(VI) (Figure 1), whereas other isolates did not grow well above 900 mg/L Cr(VI). Ilias and Camargo has been reported similar results in chromium resistant bacteria that can tolerate or reduce Cr(VI) at concentrations of 500–2000 and 1500–2500 mg/L, respectively [12,19]. The MIC of hexavalent chromium for NA2 is around 7400 mg/L; about 1000 mg/L above the concentration of the sample taken from wastewater from auto parts chromate processes (6400 mg/L). 

The isolated yellow pigmented colonies were subcultured on Luria Bertani (LB) medium and the selected colonies were then grown on MacConkey agar medium and blood agar throughout the study. The morphological and biochemical characteristics of Cr(VI) resistance strain are described in Table 1.

**Figure 2:**
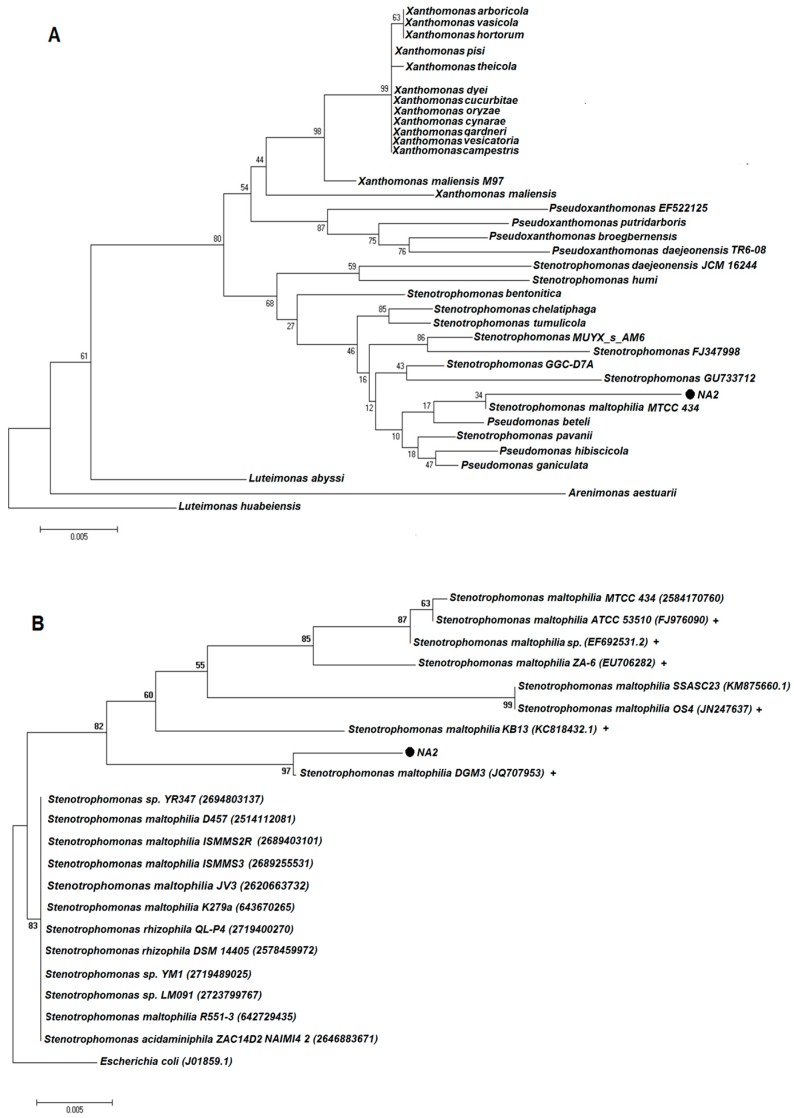
(**A**) Phylogenetic analysis resulting from the multiple alignment of 16S rRNA gene sequence of NA2 with those of other especies of the family Xanthomonadaceae found in the EzBioClould database; (**B**) Neighbor-joining phylogenetic analysis resulting from the multiple alignment of 16S rRNA gene sequence of NA2 with those of other bacterial strains of the genus *Stenotrophomonas* found in the GenBank and JGI database. The accession numbers of the strains are given in brackets. The scale bar corresponds to 0.05 substitution per nucleotide position. Experimental evidence (+).

The bacterial isolation was genotypical confirmed by polymerase chain reaction (PCR). The 16S rRNA sequence was compared with the sequences recorded in the EzBioClould database (https://www.ezbiocloud.net/). Two phylogenetic trees were constructed. The first tree was built with species of the Xanthomonadaceae family that according to the EzBioCloud database they showed a percentage of identity greater than 94%. The second tree was built with species of the genus *Stenotrophomonas* having been experimentally reported as resistant and/or reducing hexavalent chromium or that in their genome they presented the *ChrR* gene. This last criterion was validated through the JGI database (https://img.jgi.doe.gov/), using the keywords: chromate reductase and *Stenotrophomonas*. The 16S rRNA gene sequence analysis revealed that strain NA2 shows the highest level of sequence similarity with *Stenotrophomonas maltophilia* strain: DGM3 (98.01%) (JQ707953) as shown in Figure 2.

*Stenotrophomonas maltophilia*, belonging to the subclass of g-b-proteobacteria, previously known as *Pseudomonas maltophilia* and later *Xanthomonas maltophilia* [29,30], has been described in the last decades as an environmental globally emerging Gram-negative multi-drug-resistant organism that is commonly associated with diverse infections in humans such as pneumonia, bacteremia, meningitis, endocarditis, catheter-related bacteremia/septicemia and acute exacerbations in patients with cystic fibrosis and chronic obstructive pulmonary disease [31,32,33].

*Stenotrophomonas maltophilia* are naturally resistant to many broad spectrum antibiotics such as cephalosporins, carbapenems, and aminoglycosides. This means that treatment options are relatively limited, Its intrinsic resistance is particularly due to the presence of broad-spectrum efflux pumps, enzymes such as L1 metallo-b-lactamase, L2 Ambler class A b-lactamase and AAC(6′)-Iz and APH(3′)-IIa aminoglycoside modifying enzymes [34]. However novel resistance genes can be acquired by integrons, transposons and plasmids. According to the World Health Organization (WHO), *S. maltophilia* is one of the leading drug-resistant pathogens in hospitals worldwide (WHO; Public health importance of antimicrobial resistance) [31] considered as a ‘newly emerging superbug’.

In our study, the bacterial isolate NA2 showed was resistant to antibiotics amikacin, aztreonam, gentamicin and meropenem. The rate of multiple antibiotic resistance (MAR) was determined had an index 0.44, indicating that the bacterium has multiple resistance. On other hand, the isolation of *Stenotrophomonas maltophilia* (NA2) produced biofilm under the conditions evaluated (with 100 mg/L of Cr(VI) and without Cr).

However, a greater formation of biofilm in the presence of chromium was obtained, giving this bacterium the ability to survive in hostile environments with high concentrations of salts and heavy metals [35] report similar data in isolates of *Pseudomonas aeruginosa*, *Micrococcus* sp. and *Ochrobactrum* sp. resistant to chromium, attributing these facts as production of exopolysaccharides, as well as the production of aggregative fimbria, evaluated by the Congo Red test. Nancharaiah [36] indicated that bacteria in the form of biofilms may be more suitable for the Cr(VI) removal than planktonic cells, not only for their high Cr(VI) tolerance, but also for its good settlement. Only a few studies have reported the Cr(VI) removal by biofilms and suggested that biofilms of *Pseudomonas putida*, *Arthrobacter viscosus*, or mixed microbes from activated sludge possess the remarkable capacity of Cr(VI) reduction and immobilization [36,37,38]. Moreover, different bacterial systems exhibit a wide diversity of Cr(VI)-microbe interaction mechanisms due to the variety and complexity of Cr(VI)-tolerant or Cr(VI)-reducing bacteria [39].

Basu [40], reported that multiple antibiotic resistance is correlated with high degree of resistance to different heavy metals. *S. maltophilia* is able to grow at high concentrations of heavy metals, thus being resistant also to different antimicrobial agents, which suggests that the acquisition of resistance to metals and antimicrobials can occur in the natural environment from which these environmental strains are isolated [41]. However, the genomes show also sequence variability, associated to normal evolution (mutation frequency) or induced in some situations. Antibiotic pressure increases the sequence variability in resistance or related genes, as regulators. 

Other studies have shown that *S. maltophilia* has different operons involved in the import, storage and flow out of different metals including Cr(VI). Some of these metal resistance genes cluster is conserved at the same relative chromosomal position in different strains, others may appear as unique segments on specific genomic islands (GEI), highly variable. The environmental strains of *S. maltophilia* present a unique region to the operons of resistance to Hg, Co, Zn and Cd in comparison with the clinical isolates [42].

**Figure 3:**
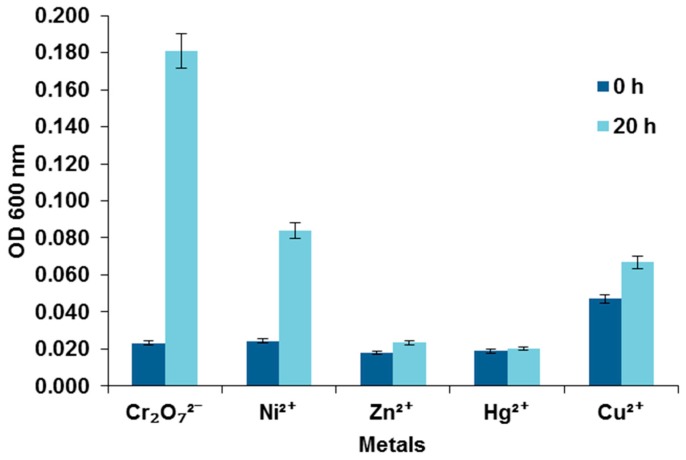
Effect of different metals on the growth of bacterial isolation.

The strain *S. maltophilia* (NA2) in this study was able to grow in the presence of Cr(VI) and Ni^2+^, this is explained by the nature of the sample, because it was taken from companies that perform nickel and chrome plating processes of auto parts (Figure 3). On the other hand, a slight growth in the presence of Zn^2+^ and Cu^2+^ was observed but not for Hg^2+^, which may suggest a natural predisposition towards the tolerance of this metal or absence of the metal resistance genes in the operon in our strain, as it has been reported for *S. maltophilia* K279a. Other explanation for the observed absence of resistance among strains could lie in the non-availability of metals and antibiotics due to the presence of poorly degradable organic compounds entrapping metals and antibiotics as reported by Oves et al. [27].

Holmes et al. [28] showed that *S. maltophilia* is able to reduce and tolerate different concentrations of Cr(VI) and is resistant to different metals such as: Cd, Hg, Au, Ag, Se and Pb as compared with *Enterobacter* sp. and *E. coli* isolated from sites contaminated with heavy metals. These results are like those of another recent report [43] showing the tolerance of *S. maltophilia* to metals such as: Ba^2+^, Cu^2+^, Fe^3+^, Mn^2+^, Ni^2+^, Ca^2+^, Mg^2+^ and Na^+^. In [44] the MIC of the chromium resistant isolates to various heavy metals was compared and it was reported that different isolates exhibited different level of metal tolerance. The tolerance to heavy metals can induce the development of mechanisms to metabolize and reduce the concentration of these pollutants in the environment.

Various mechanisms of Cr(VI) resistance or detoxification have been described, such as the efflux of chromate ions from the cell cytoplasm, reduction of extracellular Cr(VI) to Cr(III) [45], activation of enzymes involved in the ROS detoxifying processes (e.g., catalase, superoxide dismutase), repair of DNA lesions by SOS response enzymes (RecA, RecG, RuvAB), and regulation of iron uptake to prevent the production of hydroxyl radicals through the fenton reaction [46].

Chromate resistance determinants (CRDs) have been identified in Archaea, Bacteria and Eukarya, and consist of genes belonging to the chromate ion transport (CHR) superfamily [47]. Generally, CRDs include the *ChrA* gene, which encodes a putative chromate efflux protein driven by the membrane potential [46]. In bacteria, the *ChrA* genes can be located on plasmid or chromosomal DNA or both [48], and they are generally organized in operons with other *ChrR* genes. *ChrA* orthologs display highly variable resistance capacities to different Cr(VI) concentrations indicating that the only presence of the *ChrA* gene cannot explain by itself the Cr(VI) resistance and a strong activation of the Chr efflux pump could lead to the coextrusion of sulfate.

Some research has shown that Cr(VI) resistance is independent from Cr(VI) reduction which is considered a chromate detoxification mechanism and is usually not plasmid-associated [49]. Three Cr(VI) reduction mechanisms have been described: (1) Cr(VI) is reduced under aerobic conditions commonly associated with soluble chromate reductases that use NADH or NADPH as cofactors [50]; (2) Cr(VI) can be used as an electron acceptor in the electron transport chain under anaerobic conditions by some bacteria [51]; and (3) Cr(VI) can be also reduced indirectly by nonspecific reactions associated with redox intermediate organic compounds such as amino acids, nucleotides, sugars, vitamins, organic acids or glutathione [52].

**Figure 4:**
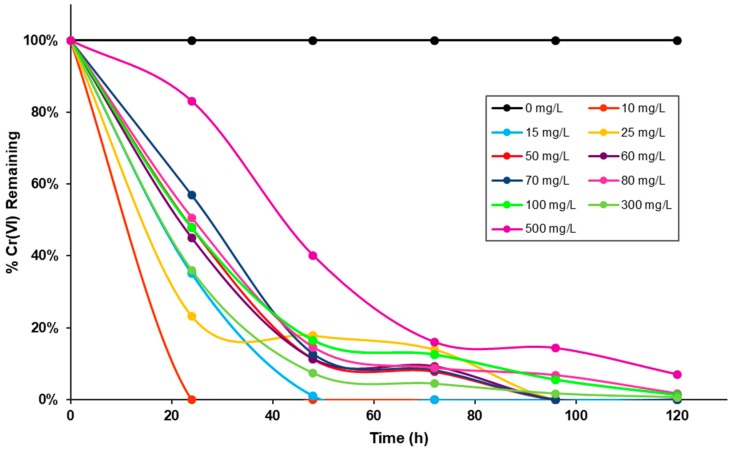
Reduction of Cr(VI) by *Stenotrophomonas maltophilia* (strain NA2). The cells were cultured on Luria–Bertani broth supplemented of 0, 10, 15, 25, 50, 60, 70, 80, 100, 300 and 500 mg/L Cr(VI). The Cr(VI) reduction activity was measured after incubation for 0, 6, 12, 18, 24, 48, 72, 96 and 120 h at 37 °C.

In Figure 4 is depicted a graphical representation of the density of the bacterial isolation and % Cr(VI) reduction at different time intervals and different concentrations. In this study *Stenotrophomonas maltophilia* (strain NA2) showed higher growth in the range of 16 to 24 h in the different concentrations of Cr(VI) evaluated (10–500 mg/L). The difference in percentage reduction between different treatments were statistically different and significant (*p* < 0.05) as determined by the one-way Analysis of Variance (ANOVA) followed by (post hoc) Tukey’s Test, a greater Cr(VI) reduction at 72 h of growth, superior 85% was observed in all cases. These results are like those described for some strains of the genus *Stenotrophomonas* reported as resistant and reducing hexavalent chromium such as *Stenotrophomonas maltophilia* OS4 [27], *Stenotrophomonas* sp. [26], *Stenotrophomonas maltophilia ZA-6* [26] and *Stenotrophomonas* sp. *JD1* [53], which presented very high percentages of reduction of Cr(VI) of 91%, 87%, 100% and 70% respectively, in periods less than 96 h.

This strain exhibited concentration dependent Cr(VI) reduction. Reducing chromium (VI) at concentrations ranging from 10 mg/mL to 70 mg/mL was 100%, while concentrations from 80 mg/mL to 300 mg/mL reduced by 98–99% and concentration of 500 mg/mL the strain reduced 92%. In non-inoculated controls the Cr(VI) reduction was negligible amount. These results are similar to those reported by Thacker [54] he has associated the decrease in chromium reduction potential of bacterium at higher concentrations to the mutagenic and toxic effects of Cr(VI) on bacterial cell metabolism.

Some number of methods focused on transforming Cr(VI) into Cr(III) have been used to decrease the toxicity of media containing Cr(VI). Chemical methods, including adding lime, coagulation, ion exchange, membrane separation, and adsorption followed by chemical precipitation as Cr(OH)_3_, have conventionally been used [44,45]. However, these processes suffer from a number of problems, including high costs, low efficiencies, and the generation of toxic sludges and other wastes that also require careful disposal and require complex operational procedures [46].

Usually, the biological species used in the bioreduction process are indigenous species which do not need any extra nutrients for their growth when they are used in the scale up applications. This is an attractive strategy that is cost-effective, safe and produces no secondary by-products. Nevertheless, when conducting a study for bioremediation the first genomic tool is investigated 16S rRNA gene sequencing that is applied to identify the organism and the next approach is genomic analysis to identify the enzymes involved in the process.

Although several studies have been carried out in which the capacity of strains of *S. maltophilia* to reduce Cr(VI) has been demonstrated, the information corresponding to the identity and characterization of the enzymes is almost null. The advance in molecular biology tools, total genome sequencing methods, comparative genomic and transcriptomic approaches attribute reductase activity of this bacterium to enzymes such as: NADPH-dependent FMN reductase, reported for *S. maltophilia* K279a (Access code: CAQ46646.1) [47]; chromate reductases reported for *Stenotrophomonas* sp. SC-N050 (access code: SET63431.1); chromate reductase *Stenotrophomonas* sp. YR347 (access code: SNS44878.1) or multispecies chromate reductase for γ-proteobacteria (reference: WP_016498740.1). These enzymes are associated with many cellular biological processes including reduction, oxidation, monooxygenation, nucleotide biosynthesis, protein folding, apoptosis, axon guidance and chromatin remodeling.

To date it has been reported that the reducing activity of hexavalent chromium in aerobic strains is due to the action of cytosolic enzymes dependent on NAD(P)H, whereas, in anaerobic bacteria, the activity is conferred by membrane components. A preliminary screening was done in this study, demonstrated the cytosolic nature of the reductase enzyme. The specific activity of chromate reductase ranged between 0.2356 to 0.8362 and 0.314 to 6457 μmol Cr(VI) reduced/min/mg protein in the cell lysate and supernatants fraction respectively. Our results contrast with those reported by Blake [55], who determined that the reduction of Cr(VI) in *S. maltophilia* was due to the activity of a membrane-associated reductase enzyme, which is expressed constitutively.

**Figure 5:**
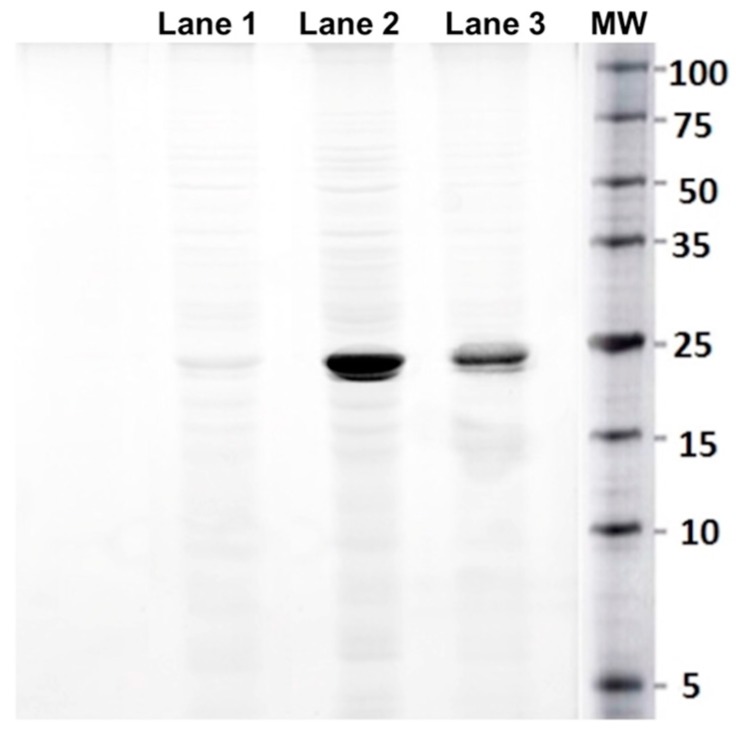
SDS-PAGE gel prepared from cytosolic protein *Stenotrophomonas maltophilia* (Strain NA2): Lane 1. Native strain exposed to 0 mg/L of chromium VI. Lane 2. Native strain exposed to 500 mg/L of chromium VI. Lane 3. Native strain exposed to 100 mg/L of chromium VI. Lane 4. Protein molecular weight marker.

Nevertheless, In Figure 5 it is shown that when comparing the profiles at different concentrations (0, 100, 500 mg/L), a protein of molecular weight around 25 kDa is induced on SDS-PAGE gel in the presence of chromium. When correlating the amount of extracted protein with the concentration of Cr(VI) of the culture medium, through a chi-square test (alpha 0.01), it was found that the concentration of proteins in the fractions depends on the concentration of chromium hexavalent in the medium, with a *p*-value of 0.0001. This could be due to the presence of chromium in the medium stimulated to the synthesis of reducing proteins in *S. maltophilia.* These results are similar to reported for the species *Bacillus* sp. *JDM-2-1*, *Staphylococcus capitis* and *Alishewanella* sp. *WH16-1* [56,57] for the reductase chromate.

Genome sequencing has revealed much of the genome to be conserved across different *S. maltophilia* strains [42]. In silico analysis validated through the JGI database (https://img.jgi.doe.gov/) in this contribution, using the keywords: chromate reductase and *Stenotrophomonas,* revealed the presence of the *ChrR* gene in 12.4% of 89 genomes reported with a higher prevalence in the species *S. maltophilia*, followed by *S. rhizophila* and *S. acidaminiphila*.

Aguilar et al. [58] demonstrated in strains of *P. aeruginosa* and *Shewanella* sp. that the *ChrR* gene plays an essential role in the function of ChrA, since when this gene is altered, ChrA no longer confers resistance to chromate. Possibly, *ChrR* regulate the genes involved in the reduction of Cr(VI) and detoxification that are necessary for chromate resistance conferred by ChrA.

To date few studies have been are recognized to purification and characterization of Cr(VI) chromate reductases, only *P. putida* (ChrR), *E. coli* (YieF) and *Alisshewanella* (ChrR) has been reported [50,53,56,57]. ChrR catalyzes a combination of one- and two-electron transfers to Cr(VI) with the formation of the unstable species Cr(V) before further reduction to Cr(III), evidenced the Type II “semi-tight” mechanism Cr(VI) of reduced in two steps; the first step involves one electron transfer forming Cr(V) with another electron donated to oxygen forming ROS and second step involves two electrons transfer to Cr(V) to form Cr(III). The resultant intermediates can cause oxidative damage to proteins and DNA by forming a range of DNA lesions, together with Cr-DNA adducts, DNA-DNA crosslinks, DNA-protein crosslinks [4,49,59]. Although a proportion of the Cr(V) intermediate is spontaneously reoxidized to generate ROS continuously, its reduction to Cr(III) through two-electron transfer, minimizes the production of harmful radicals. With the continued activity of the one-electron reducers, chromate shuttles back and forth between its Cr(VI) and Cr(V) valence states, producing large quantities of ROS and depleting the cell’s reducing power. The oxidative stress affects viability of cells and the efficiency of Cr(VI) reduction. Thus, ROS generated by ChrR activity during Cr(VI) reduction should be neutralized by quinols formed by the quinone reductase activity of the same enzyme [4,45,60].

In contrast, YieF of *E. coli* directly reduces Cr(VI) by transferring four electrons, exhibited the Type I “tight” mechanism involves one step transfer of three electrons from dimeric flavoenzymes to Cr(VI) with one electron transfer to oxygen resulting in ROS [60].

**Figure 6:**
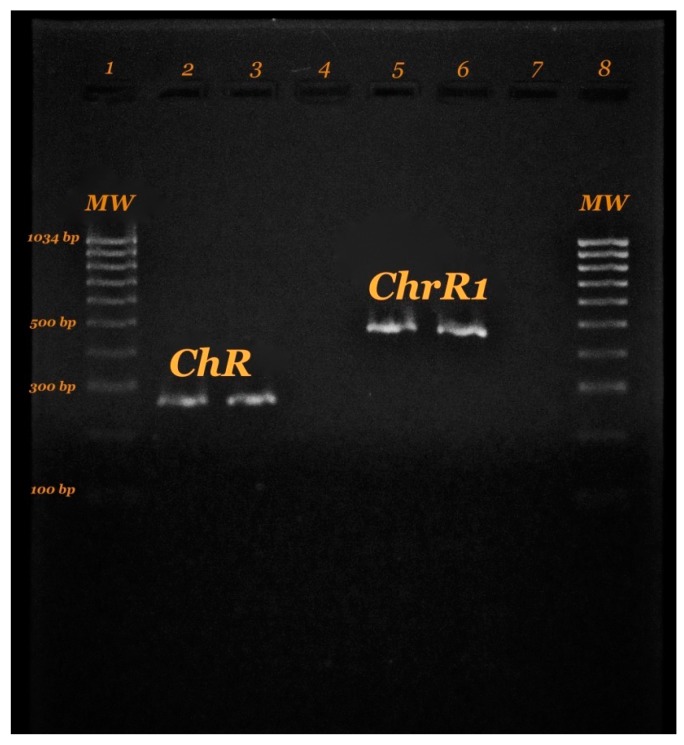
Chromate reductase in this study. Lane 1. 100 bp DNA Ladder-Thermo Fisher. Lanes 2–3. Gene positive *ChR* Patra et al. Lane 4. Negative Control. Lanes 5–6. Gene positive *ChrR1* (Designed this study). Lane 7. Reactive Control. Lane 8. 100 bp DNA Ladder-Thermo Fisher.

In this contribution, we demonstrated that the presence of chromate reductase gene in *S. maltophilia,* a fragment of around 468 bp was obtained using the primers designed in this study. Likewise, an amplicon of 268 bp was obtained according to the previous protocol reported by Patra [61], to partially amplify the *ChrR* gene for Gram positive isolates *Arthrobacter aurescens*, *Bacillus atrophaeus* and *Rhodococcus erythropolis* (Figure 6). This gene is considered as a genetic determinant highly like phylogenetically distant microorganisms.

This study constitutes for the best of our acknowledge the first report that experimentally demonstrates the identity of the genes involved in Cr(VI) reductase, and its expression in *Stenotrophomonas maltophilia*. In this sense, primers used in our study designed from complete genome sequence of *Stenotrophomonas maltophilia* K279a (GenBank accession No. NC_010943), could serve as an effective tool to identify organisms either in culture or in bacterial consortium of having Cr(VI) reducing genetic potential for its exploitation in Cr(VI) bioremediation by its reduction to Cr(III).

Among the chromate reductases those forming less ROS are suggested for bioremediation since the viability of detoxifying cells is not affected. The quinone reductase activity of chromate reductase improves tolerance to peroxide. When the chromate reductase was over expressed in remediating cells, it not only increased the rate of Cr(VI) reduction but improved cell viability by minimizing oxidative stress. The results indicate the possibility of employing chromate reductases of this kind for development of bacteria for bioremediation. The presence of other co-pollutant metal ions in contaminated sites would affect the bioremediation potential of chromate reductases. Engineering chromate reductase to improve tolerance to other metal ions is inevitable. Barak et al. [62], have shown that mutation of chromate reductase by directed evolution markedly improved the capacity of reduction of two toxic metal ions Cr(V) and U(VI).

Some *S. maltophilia* strains are also known for their biotechnological potential as they can contribute to bioremediation and phytoremediation strategies [41,63] and to the production of biomolecules of economic value [64]. The capacity of *S. maltophilia* has been demonstrated in various processes such as bioremediation of environments contaminated by pesticides as chloropyrifos and endsulfan, surface disinfection, reduction of hydrocarbons and heavy metals such as selenium, copper, chromium and lead biosorption, zinc, nickel by turning the genes on and off as the organism detects something appetizing [8,65]. The great genetic and metabolic diversity within *S. maltophilia* makes it a “Wonder-bug”.

**Figure 7:**
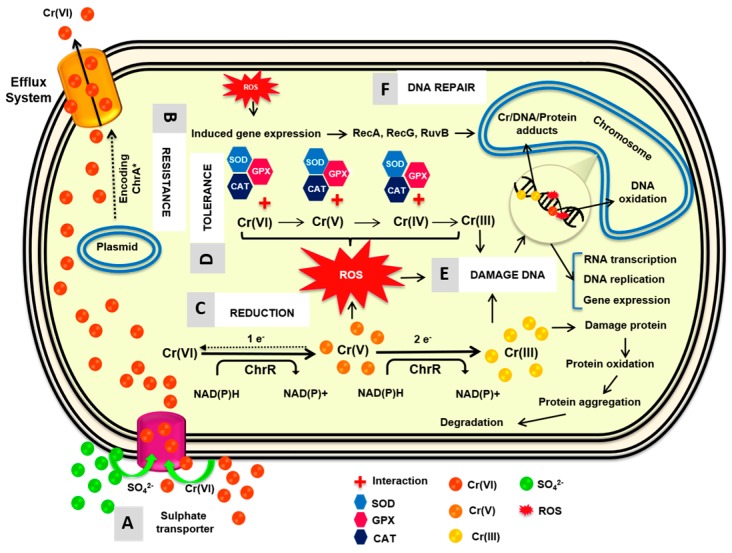
Possible mechanisms of interaction of *Stenotrophomonas maltophilia* and chromate. **A**. Chromate enters the bacterial cell through sulfate transporter. **B**. Resistance: Plasmid DNA encoded efflux systems. * This system in some bacteria is ChrA transporter. **C**. Intracellular reduction of Cr(VI) to Cr(III) involves soluble chromate reductase ChrR which requires NAD(P)H as an electron donor. **D**. Tolerance: To combat the ROS generated oxidative stress, protective metabolic enzymes superoxide dismutase, catalase and glutathione are secreted. **E**. Damages: Cr(VI) and Cr(III) negatively affects DNA, RNA and proteins. **F**. SOS system: DNA repair system. Modified from [8,43,66].

In this work it was possible to demonstrate the role of indigenous *S. maltophilia* in environments contaminated by chromium VI and able to make enzymes that play vital role in bioreduction process. Based on the results obtained and on the similarity of the chromate reductases, it is possible to infer the following reduction mechanism (Figure 7).

## 3. Materials and Methods

### 3.1. Collection of Effluents

Effluent samples were collected in sterilized bottles from automobile part chrome plating processes in Cartagena (Colombia). Some physicochemical parameters of the wastewater such as temperature (°C), pH, dissolved oxygen and chromium (μg/mL) were measured [67]. Samples were brought to immediately to the laboratory in an icebox, stored at 4 °C and used as inoculum source to obtain isolates capable of removing Cr(VI) under aerobic conditions.

### 3.2. Bacterial Strains and Cultivation Conditions

The bacterial strains were isolated using a culture enrichment technique. The microorganisms were enriched in 90 mL Luria Bertani Broth (LB) medium amended with K_2_Cr_2_O_7_ at 100 mg/L and 10 mL of effluent samples, the mixture was incubated a 37 °C by shaking at 200 rpm for 24 h. 

The enriched culture was isolated on LB agar plates amended with K_2_Cr_2_O_7_ at concentrations of 100, 300, and 500 mg/L, cultured for 7 days at 37 °C. The isolates which showed a color change from orange to grey green during growth were regarded as Cr(VI) reducing bacteria [68].

Strains that could grow on a LB plate containing K_2_Cr_2_O_7_ at 500 mg/L were selected for further studies. Individual colonies of different morphologies were picked up and purified by repeated transferring onto the medium supplemented with Cr(VI), until obtaining pure cultures confirmed by microscope [69]. Subsequently, the effect of varying concentrations (10–2000 mg/L) of Cr(VI) on the tolerance of the microbial growth was examined in triplicate in LB broth (5 mL) at 37 °C under 200 rpm in a water bath for 72 h. The growth was monitored measuring OD at 600 nm, using glucose as the sole carbon source (0.5% *w*/*v*).

Single colonies of selected strains were sub-cultured onto fresh LB agar medium and glycerol stock was prepared by mixing equal amounts of bacterial culture (Trypticase soy broth) and 80% glycerol (giving a final 40%) stored at 80 °C. Out of the total 13 bacterial strains isolated, and designated as NA1 at NA13, were recovered. The isolate that showed the highest Cr(VI) resistance (NA2) was selected for further studies.

### 3.3. Identification of Selected Bacterial Isolate

Strain NA2 was identified by morphological and biochemical parameters for standard method [70]. The molecular characterization of the isolate was done by 16S rRNA gene sequencing [71].

#### 3.3.1. Morphological and Biochemical Identification

Morphological observations were made from pure cultures of the isolated strain grown on LB medium agar at about 37 °C under ambient conditions of diffuse daylight. Each isolate was examined for its size, shape, margin, consistency, elevation, pigmentation, Gram reaction and cell morphology. The isolates were characterized as described by Bernardet et al. [72].

The isolated colonies were identified by routine biochemical tests. A presumptive identification was performed by the following tests: Gram staining, catalase test, oxidase test, urease test, indole test, methyl red-Voges Proskauer (MR-VP) test, citrate test, starch hydrolysis, triple sugar iron (TSI) test, gelatin hydrolysis, casein hydrolysis test, decarboxylation test, nitrate reduction test, phenyl alanine deamination, DNase Test, chitin hydrolysis, lipase test, esculin hydrolysis, among others.

#### 3.3.2. Molecular Characterization

##### DNA Extraction

DNA was extracted to according to previously published work by Sambrook et al. [73]. All isolates were prepared by inoculating a single colony into 1 mL of LB broth and incubated at 37 °C with shaking (~100 rpm) overnight. Samples were washed and resuspended in 10 mM Tris, 1 mM EDTA (TE) buffer before addition of 50 μL of 10% (*w*/*v*) filtered sodium dodecyl sulfate (SDS) and 50 μL of proteinase K (2 mg/mL stock solution). Samples were shaken at 37 °C until clear (1 h). DNA was purified by sequential phenol: chloroform: isoamyl alcohol extraction and ethanol precipitation. The final DNA concentration was measured with a NanoDrop spectrophotometer (NanoDrop™ 2000, Thermo Scientific, Waltham, MA, USA) [74]. The extracted DNA was electrophoresed on 1% agarose gel in TBE buffer and visualized under UV (Gel Doc, Bio-Rad Laboratories, Hercules, CA, USA) to check for integrity. DNA was stored at 20 °C until further use.

##### Amplification of 16S rRNA Gene

The 16S rRNA gene was amplified using the universal primers 27F (5′ AGAGTTT-GATCMTGGCTCAG 3′) and 1492R (5′ GGTTACCTTGTTACGACTT 3′) [70]. PCR reactions were performed in a final volume of 20 μL, containing 1 μL of DNA (50 ng/μL), 1× buffer 2 μL, 3 mM of MgCl_2_, 0,2 mM of each dNTP, 1U of Taq polymerase (Fermentas, Waltham, MA, USA), 0.4 μM of each of forward and reverse primers and deionized water to make the volume.

PCR amplification was carried out in a cycler thermalcycler (T100™ Thermal Cycler Bio-Rad) subjected to denaturation at 95 °C for 5 min, followed by 26 cycles consisting of denaturation at 94 °C for 45 s, annealing at 56 °C for 45 s and extension at 72 °C for 90 s and a final extension of 72 °C for 10 min. The PCR products were separated on 1.5% agarose gels in TBE 1× buffer stained with ethidium bromide (0.5 mg/mL) and visualized with UV light. The PCR products were purified using the QIAquick PCR Purification Kit (Qiagen, Hilden, Germany), according to the manufacturer’s instructions and sequenced commercially by Sequencing Service, Macrogen Inc., Seoul, South Korea.

##### Analysis Sequence

The chromatogram sequencing files were edited using Chromas software (version 2.6.4, Technelysium, South Brisbane, Australia). 16S rRNA gene sequences were assembled with SeqMan Pro 13 free trial version (DNASTAR, Madison, WI, USA). The homology of the 16S rRNA gene sequences was checked with the 16S rRNA gene sequences of other organisms that had already been submitted to GenBank database using the BlastN (http://www.ncbi.nih.gov/BLAST/) algorithm. Then, the inconsistencies or conflicts found in the Editseq software were eliminated. For the identification of the isolated bacteria, consensus sequence was inserted into EzBioCloud database (https://www.ezbiocloud.net/), where the strain of the library was chosen that showed the greatest similarity.

##### Phylogenetic Tree Construction 

The phylogenetic tree was constructed using the neighbour-joining method and the Kimura 2-parameter model of distance analysis, and 1000 bootstrap replications were assessed to support internal branches [75]. Similitude analysis was estimated using nucleotide sequences with MEGA 7 [76]. *E. coli* K12 was taken as out-group. Phylogenetic affiliations were based on the limits proposed by Rosselló and Amann [77], where <95, 95–97.5 and >97.5% define the taxonomic levels of family, genus and species respectively.

#### 3.3.3. Antibiotic Susceptibility Testing

Bacterial susceptibility to antimicrobial agents was determined according to the manufacturer’s recommendations by overnight microdilution method with commercial dehydrated panels (NUC 60) provided by Dade Behring MicroScan (Sacramento, CA, USA) that were read by the autoSCAN-4 and interpreted according to Clinical Laboratory Standards Institute (CLSI) guidelines [78]. The following antibiotics were used: ampicilline (AMP), ampicillin/sulbactam (SAM), cefotaxime (CTX), nitrofurantoin (NIT), amikacin (AMK), ciprofloxacin (CIP), trimethoprim/sulfametoxazole (SXT), aztreonam (AZM), cefazolin (CEZ), cefepime (FEP), cefoxitin (FOX), ceftazidime (CAZ), Ceftriaxone (CRO), doripenem (DOR), ertapenem (ETP), gentamicin (GEN), meropenem (MEM), piperacilin/tazobactam (TZP), piperacilin (PIP) and tobramycin (TOB). Multidrug resistance (MDR) was defined as non-susceptibility to at least one agent in three or more antimicrobial categories [79].

### 3.4. Microtiter Plate Assay (Quantitative Assays for Biofilm Formation)

A crystal violet staining method was employed to examine biofilm-forming abilities of the isolates [80] with some modifications. Bacteria were first grown overnight in LB broth 0.25% glucose with or without 100 mg/mL chromium VI. The isolates were inoculated into 1 mL LB broth and grown overnight at 37 °C with constant shaking. Overnight, cultures were transferred to new culture medium (diluted by 1:100) and grown to OD 600 between 0.45 and 0.65 and for each strain assay, it was done in triplicate. Thirty microliters of bacteria in log phase growth were inoculated into 96-well polystyrene plates containing 100 mL fresh LB broth and incubated at 37 °C for 24 h. The plates were rinsed three times with deionized water and the adherent bacteria cells were stained with 0.5% crystal violet for 30 min. After being rinsed three times with deionized water, the crystal violet was liberated by 80% ethanol and 20% acetone following a 15 min incubation. The OD values of each well were measured at 492 nm. The tested strains were classified according to the criteria of Stepanovic et al. [81] into non-biofilm producer (OD ≤ ODc), weak biofilm producer (OD > ODc, but ≤2 × Dc), moderate biofilm producer (OD > 2 × ODc, but ≤4 × ODc), and strong biofilm producer (OD > 4 × ODc).

### 3.5. Minimum Inhibitory Concentration (MIC)

Minimum inhibitory concentration (MIC) of Cr(VI) for the isolates was determined on LB plates supplemented with filter sterilized Cr(VI) at different concentrations (100 mg/L to 10,000 mg/L) and glucose as the sole carbon source (0.5% *w*/*v*) [82]. After incubation at 37 °C, for 5 days, the highest concentration of Cr(VI) which permitted growth and beyond which there was no growth was considered as MIC of Cr(VI) for the isolates tested.

### 3.6. Growth Tolerance and Chromium (VI) Reduction

Growth tolerance and Cr(VI) reduction assay was performed by growing the isolate in 50 mL LB broth supplemented with 0, 10, 50, 100, 300, 350, 400, 450, 500, 1000 and 2000 mg/L of Cr(VI). A 0.5 mL aliquot of the original culture (OD 600 = 1.0 ± 0.05) was transferred to each of a series of 50 mL aliquots of LB medium and the mixtures were incubated at 37 °C, pH 7.0 with shaking at 200 rpm for 120 h to investigate the abilities of the isolates to reduce Cr(VI) [17,82]. A 0.5 mL aliquot of each mixture was withdrawn every 24 h and then divided into two portions [83,84,85]. 

One portion was centrifuged at 10,000 rpm for 10 min and supernatants were analyzed for residual Cr(VI) concentration, determined using the 1,5-diphenylcarbazide (DPC) method [15,86]. Carbazide reagent was prepared in acetone/H_2_SO_4_ to minimize deterioration [87] as follows: DPC (0.025 g) was dissolved in 10 mL acetone, 3M H_2_SO_4_ was used to acidify the samples. The carbazide reagent was stored at 4 °C until use.

The reaction mixture was set up in an Eppendorf tube containing the following: 200 μL sample or standard sodium chromate solution and 400 μL solution (136 μL 3M H_2_SO_4_ and 264 μL 0.25% (*w*/*v*) DPC). Spectrophotometric measurements were made immediately at 540 nm. The presence of a purple color indicated when hexavalent chromium is present. Cr(VI) concentration in the sample was quantified using a standard plot prepared from K_2_Cr_2_O_7_.

The percent reduction of Cr(VI) was calculated using the formula: [(Ci − Cf)/(Ci × 100), where, Ci 1/4 initial Cr(VI) conc. (mg/L) and Cf 1/4 final Cr(VI) conc. (mg/L)] [88]. The uninoculated LB broth containing 200 mg/L Cr(VI) was served as negative control. All tests were performed in three replicates. Another portion was used to evaluate cell growth by measuring optical density at 600 nm. A control treatment without bacterial strain inoculation was processed. All the experiments had three replicates.

### 3.7. Tolerance to Other Metals

The effect of the different metals (Hg, Ni, Zn and Cu) on the growth of bacterial isolates was examined in LB broth (5 mL) supplemented with 200 mg/L of selected metals and 50 mg/L Cr(VI). The stock solutions were prepared from the analytical grade salts HgCl_2_, NiCl_2_-2H_2_O, ZnCl_2_, CuCl_2_ for Hg(II), Ni(II), Zn(II) and Cu(II) ions. The tubes were inoculated with 25 μL of the inoculum and incubated at 37 °C for 24 h. The growth of the bacterial isolates was measured spectrophotometrically at 600 nm [12].

### 3.8. Extraction and Localization of Chromate Reductase

Subcellular fractioning was carried out according to the Ilias et al. [12] technique with some modifications. 10 mL of cell culture was prepared in LB broth supplemented with 0.1% glucose and Cr(VI) in concentrations of 0, 100 and 500 mg/L. Samples were incubated at 37 °C and 150 rpm for 24 h. 1 mL of culture was extracted from each tube and centrifuged at 9000 rpm during 15 min at 4 °C. The resulting pellet was washed 2 times with 10 mM Tris-HCl buffer pH 7.2, and suspended in 500 μL of the same solution. The suspended cultures were then disrupted by ultrasonication employing one cycle of sonication for five minutes at one-minute intervals and 50 W in cold conditions. The homogenate was then centrifuged at 14,000 rpm for 40 min at 4 °C. The supernatant or soluble fraction was stored at 4 °C. Cell pellet was re-suspended in 500 μL of 10 mM Tris-HCl, pH 7.2. The proteins were quantified by the Bradford method and the absorbance was measured using a UV-Vis spectrophotometer at a wavelength of 595 nm.

### 3.9. Polyacrylamide Gel Electrophoresis

SDS-PAGE was carried out on 10% gels as described by Laemmli [89], with dimensions of 8 cm × 8 cm and 1 mm thick. The gels were run at 125 V constant for 2 h at room temperature and stained overnight with Coomassie Brilliant Blue. To locate the protein and determine the kinetics of the same, the fractions obtained in solution of hexavalent chromium with a concentration of 2 mg/L and a negative control (without proteins) were inoculated. The samples were incubated in a water bath at 37 °C for 1 h. The Cr(VI) concentration was determined using the 1,5-diphenylcarbazide method.

### 3.10. Amplification of Cr(VI) Reductase Gene

The amplification of the chromate reductase *ChrR* gene was performed based on the primers previously reported by Patra et al. [61] and those designed in this study using the complete genome sequence of *Stenotrophomonas maltophilia* K279a (GenBank accession No. NC_010943) that target nucleotide positions 2619103–2619702 (Table 2). Primers were designed using Primer-BLAST (NCBI, Bethesda, MD, USA, https://www.ncbi.nlm.nih.gov/tools/primer-blast).

PCR amplification of Cr(VI) reducing gene was carried out in an thermalcycler (Thompson) subjected to denaturation at 95 °C for 5 min, followed by 35 cycles consisting of denaturation at 95 °C for 30 s, annealing at 62 and 58 °C (respectively) for 30 s and extension at 72 °C for 1 min and a final extension of 72 °C for 10 min.

The PCR products were separated on 1.5% agarose gels in TBE buffer stained with ethidium bromide (0.5 mg/mL) and visualized with UV light. PCR reactions were conducted in 25 μL aliquots containing 50 ng template DNA, 10 pM of each primer, Dream Taq PCR Master Mix (2×) (Thermo Fisher Scientific, Waltham, MA, USA) and water.

### 3.11. Statistical Analysis

All experiments were carried out by triplicate. The standard deviation of the mean, the Fisher’s least significant difference and the difference in percentage reduction between different treatments were determined by the one-way Analysis of Variance (ANOVA) followed by (Post Hoc) Tukey’s Test (*p* < 0.05) were analyzed through XLSTAT software version 13.0 (Addinsoft, Chicago, IL, USA).

## 4. Conclusions

Identification of the bacterial strain *Stenotrophomonas maltophilia* isolated from sites contaminated by Cr(VI) was achieved by biochemical assays and analysis of 16S rRNA gene, this bacterial strain had the ability to reduce Cr(VI) in a range of concentrations of 10 to 500 mg/L. This reduction was associated with the soluble fraction of the cell, through the extraction and localization of a protein with an approximate size of 25 kDa, in addition to the in silico analysis in this study, revealed the presence of the chromate reductase gene *ChrR*, which is associated with the protein found. In addition, the strain was a biofilm producer, multiresistant to antibiotics and other heavy metals such as: Cr, Ni, Zn and Cu, making it a good candidate for bioremediation, biosorption and/or bioaccumulation processes.

## Figures and Tables

**Table 1:** Morphological and biochemical characteristics of Cr(VI) resistance strain.

Strains *Stenotrophomonas maltophilia*						
Features	Specifications	* NA2	DGM3 ^a^	T7D7 ^b^	OS4 ^c^	ZA6 ^d^
Appeareance	Phenotypic Characteristics	* Pale Yellow, Glistening, Circular Colonies with Entire Smooth Margin. Lavender Green Colonies on Blood Agar. Straight or Curved Rods, 0.5 by 1.5 μm.				
Staining	Gram Reaction	−	−	−	−	−
Biochemical reactions	Motility	+	+	+	+	+
	Methyl Red	−	v	−	−	−
	Citrate	+	+	+	−	−
	Nitrate	+	v	v	v	v
	Indole	−	−	−	−	−
	Catalase	+	+	+	+	+
	Voges Proskauer	−	−	−	−	−
	Oxidase	−	−	−	−	−
	Urease	−	v	v	+	−
	Dnase	+	v	v	+	+
Hydrolysis	Starch	+	v	−	v	v
	Gelatin	+	v	+	v	+
	Esculin	+	v	v	v	v
Carbohydrates utilization	Sucrose	+	v	+	+	+
	Glucose	+	v	v	v	+
	Mannitol	−	v	−	−	−
	Sorbitol	−	v	−	−	−
	Lactose	+	v	−	−	+
	Florescence (UV)	+	−	v	v	v
	H_2_S Production	−	v	v	v	v

+ Reaction positive. − Reaction negative. * Data in this study. ^a, b, c, d^ Data from Gunasundari et al. [26] (JQ707953), Oves et al. [27] (JN247637), Holmes et al. [28] (FJ976090) and Alam and Ahmad [21] (EU706282) respectively. v Data not shown for reference.

**Table 2:** Primers used for amplification of chromate reductase.

Gene	Sequence Primers/Probe	Genome	Size Fragment	References
*ChrR1F*	5′ AGCAACAGTTACAACCGGC 3′	Chromate reductase	458 bp	Designed in this study
*ChrR1R*	5′ CGAAATCTTCCGCGTCATCG 3′			
*ChRF*	5′ TCACGCCGGAATATAACTAC 3′	Chromate reductase	268 bp	[61]
*ChR*	5′ CGTACCCTGATCAATCACTT 3′			
